# Health in the 2030 Agenda for Sustainable Development: from framework to action, transforming challenges into opportunities

**DOI:** 10.7189/jogh.09.020201

**Published:** 2019-12

**Authors:** Dominik Dietler, Andrea Leuenberger, Nefti-Eboni Bempong, Diarmid Campbell-Lendrum, Conradin Cramer, Rik I L Eggen, Séverine Erismann, Silvia Ferazzi, Antoine Flahault, Helen A Fletcher, Bernhard Fuhrer, Samuel Fuhrimann, Helena Greter, Anne Christine Heerdegen, Melissa Leach, Anna Leissing, Jonathan Lilje, Melissa A Penny, Helen Prytherch, Philipp Staudacher, Penelope Vounatsou, Frederik Weiss, Ruth Wiedemann, Mirko S Winkler, Xiao-Nong Zhou, Jürg Utzinger

**Affiliations:** 1 Swiss Tropical and Public Health Institute, Basel, Switzerland; 2 University of Basel, Basel, Switzerland; 3 Institute of Global Health, Faculty of Medicine, University of Geneva, Geneva, Switzerland; 4 Department of Public Health, Environmental and Social Determinants of Health, World Health Organization, Geneva, Switzerland; 5 Department of Education, Basel, Switzerland; 6 Eawag, Swiss Federal Institute of Aquatic Science and Technology, Dübendorf, Switzerland; 7 ETH Zurich, Federal Institute of Technology, Zurich, Switzerland; 8 Medicines for Malaria Venture, Geneva, Switzerland; 9 United Kingdom Research and Innovation, London, United Kingdom; 10 Swiss Network for International Studies, Geneva, Switzerland; 11 Institute for Risk Assessment Sciences, Utrecht University, Utrecht, The Netherlands; 12 Institute of Development Studies, University of Sussex, Brighton, United Kingdom; 13 swisspeace, Bern, Switzerland; 14 Institute of Political Science, University of Bern, Bern, Switzerland; 15 National Institute of Parasitic Diseases, Chinese Center for Disease Control and Prevention, Shanghai, People’s Republic of China; 16 Key Laboratory of Parasite and Vector Biology, Ministry of Health, Shanghai, People’s Republic of China; * Equal contributions

The critically important role of health for development was underlined in the 16th World Development Report entitled “Investing in health”, published in 1993 [1]. Put forth by the World Bank and enhanced with input from the World Health Organization (WHO), the report examined the interplay between human health, health policy, and economic development. In the period 2000-2015, health for development was strongly emphasized in the Millennium Development Goals (MDGs). Indeed, three of the eight MDGs explicitly featured health [2]. Meanwhile, major achievements have been made in population health. For instance, average global life expectancy has increased by more than 20 years between 1950 and 2010 [3]. Yet, there are areas of unfinished business, such as reducing child mortality and improving maternal health [4]. Key vulnerable groups, such as the poorest and most isolated populations, have been left excluded and marginalized [4,5]. In addition, there are new challenges, as for instance non-communicable diseases have surpassed infectious diseases in terms of global burden [6], novel infectious threats from zoonoses [7] and anti-microbial resistance [8] have emerged, there are toxic mixtures of chemicals compromising human, animal, and ecosystem health, while climate change, urbanization, and migration have amplified health problems and vulnerabilities [9]. Taken together, there are multifactorial stresses that ask for innovative, multi-partner, integrated approaches.

With the launch of the Sustainable Development Goals (SDGs) in 2015, a more holistic agenda that unifies economic, environmental, and social aspects of development, and which is universally applicable, was adopted [10]. While health is specifically addressed in only one of the 17 SDGs, namely SDG 3 “Ensure healthy lives and promote well-being for all at all ages”, health is recognized as a cross-cutting issue that is directly embraced in 28 of the 169 SDG targets [11,12]. Moreover, other goals – such as those addressing poverty (SDG 1), hunger (SDG 2), gender equality (SDG 5), clean water and sanitation (SDG 6), reduced inequalities (SDG 10), and climate action (SDG 13) – as well as the overarching SDG principle to “leave no-one behind” will have indirect impacts on health. Hence, addressing health in the SDGs means achieving both many individual targets and a broader ‘package’ of goals. To do so in the little remaining time is a tall order. Consequently, innovative and scalable approaches are required, including novel partnerships that are guided by principles of ecological and social justice.

In November 2018, a one-day symposium jointly organized by the Basel-based Swiss Tropical and Public Health Institute (Swiss TPH) and the Geneva-based Swiss Network for International Studies (SNIS), brought together people from academia, local government, private sector, product development partnerships, funding agencies, international organizations, and civil society (**Table 1**). The variegated public presented, exchanged, and debated on the theme “Health in the 2030 Agenda for Sustainable Development”. The symposium, attended by some 125 people, consisted of key-notes, speed talks, and a documentary of the “Pesticide use in tropical settings” (PESTROP) project film [13]. The symposium programme can be found here.

**Table 1:** Exchange platforms, projects, and initiatives fostering inter- and transdisciplinary research and multisectoral collaborations that are key in the 2030 Agenda for Sustainable Development presented in a one-day symposium jointly organized by the Swiss Tropical and Public Health Institute (Swiss TPH) and the Swiss Network for International Studies (SNIS)

	Website
**Exchange platforms**	
Basel Peace Forum	https://basel-peace.org/
Ebola Response Anthropology Platform	http://www.ebola-anthropology.net/
Epidemic Response Anthropology Platform (ERAP)	https://www.epidemicresponse.net/
Social Science in Humanitarian Action	https://www.socialscienceinaction.org/
Swiss Network for International Studies (SNIS)	https://snis.ch/
UKRI Interdisciplinary Research Hubs	https://www.ukri.org/research/global-challenges-research-fund/interdisciplinary-research-hubs-to-address-intractable-challenges-faced-by-developing-countries/
UNLEASH Network	https://unleash.org/
**Projects**	
Dynamic Drivers of Disease in Africa (DDDAC)	https://steps-centre.org/project/drivers_of_disease/
Malaria Risk Map	(under development)
PESTROP	https://www.swisstph.ch/de/projects/pestrop-pesticide-use-in-tropical-settings/
Precision Epidemic Forecasting	https://www.unige.ch/medecine/isg/en/research/945flahault/

While the thematic exchange and networking was the primary focus of the event, the organizers also seized the opportunity to collect primary data among the speakers and participants in an effort to map the attendees’ insights into the complex issues of sustainable development. Data were collected through (i) a questionnaire survey that was addressed to all 23 speakers and moderators from 13 different institutions shortly before the symposium and (ii) an e-poll offered to all participants at the opening and towards the end of the symposium. Questionnaire recipients were asked to state the three most important SDGs in their daily work, and the key challenges and opportunities to reach the goals. Data from the e-poll were visualized in real-time, which further stimulated discussion and debate.

As the symposium focused on health, it is not surprising that SDG 3 featured particularly prominently among speakers and chairs’ responses to the questionnaire (**Figure 1**). This pattern was even more striking in the e-poll; two-thirds of the participants rated SDG 3 as the single most important SDG in their daily work. The second most important SDG for the speakers and chairs was SDG 17 “Strengthen the means of implementation and revitalize the global partnership for sustainable development”. Overall, 13 of the 17 SDGs were listed among the three most important SDGs, demonstrating the collective breath of interest and expertise at this symposium.

**Figure 1:**
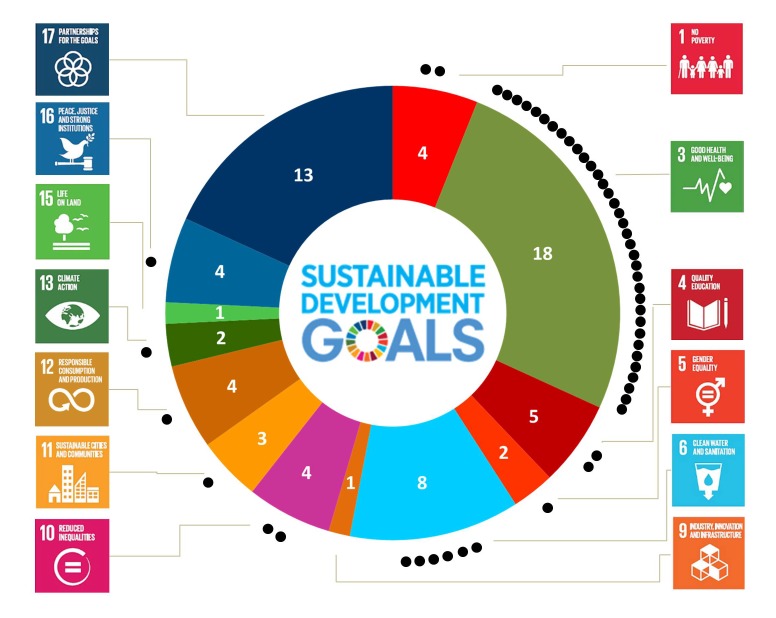
Sustainable Development Goal (SDG)-donut chart indicating the three most important SDGs among the 23 presenters and chairs (n = 23) of a one-day symposium jointly organized by the Swiss Tropical and Public Health Institute (Swiss TPH) and the Swiss Network for International Studies (SNIS), held in Basel in November 2018. The black dots represent the number of votes for the single most important SDG from the audience that participated in the e-poll at the beginning of the symposium (n = 52).

Over the course of the event, two themes emerged: (i) the interconnection of the SDGs and (ii) the need for collaboration and novel partnerships. Hereunder, four challenges became clearly visible and prompted discussions as to potential solutions. The challenges were confirmed by the e-poll at the end of the event, underlining their importance in the 2030 Agenda for Sustainable Development beyond the health-related targets.

## CHALLENGE #1: DEVELOP CONCEPTS AND PROPOSALS COLLABORATIVELY

Potential solution: It is pivotal to engage partners from different geographical regions already at the conceptual phase of a project. This practice to jointly develop the research agenda is also one of the main principles in the “Guide for transboundary research partnerships”, put forth by the Swiss Commission for Research Partnerships with Developing Countries (KPFE) [14]. The importance of mutually beneficial, equitable partnerships for sustainable development has recently been discussed and promoted by a group of experts with decades of experience in North-South and South-South collaboration [15]. However, in practice, leveraging international partnerships for sustainable development is challenging, partly because of uneven distribution of science and technology capacity in high-income compared to low- and middle-income countries, which comes with unequal access to knowledge and funding opportunities. Moreover, building the relationships that underpin successful partnerships takes time.

This international collaboration on eye-level needs urgent attention and it comes as no surprise that the 2030 Agenda for Sustainable Development stresses the need to federate existing knowledge with collaborative approaches. Specifically, SDG 17 insists that this methodological effort is crucial to address all the other goals. During the symposium two concrete examples of collaborative idea development were presented. First, the “UNLEASH innovation lab” (see **Table 1**) was introduced as an incubator bringing together actors from different places, backgrounds, and sectors to foster SDG-specific solution-finding. Second, the two funders that participated at the symposium (ie, SNIS and United Kingdom Research and Innovation (UKRI), which hosts the Global Challenges Research Fund (GCRF) and the Newton Fund) offer funding schemes, where international collaboration is mandatory to receive grants. Of note, UKRI promotes partnerships where research is co-funded through the Newton Fund. The Newton Fund engages 17 middle-income partner countries to set research priorities and jointly finance research projects in order to move towards equitable and sustainable North-South partnerships to tackle some of the most intractable development challenges.

## CHALLENGE #2: IMPLEMENTING PROJECTS WITH INTER- AND TRANSDISCIPLINARITY TEAMS

Potential solution: Fostering collaboration and joint work between academic disciplines (including across the health, natural, and social sciences) to engage in interdisciplinary work holds untapped potential. Subsequently the scope of collaboration could still be broadened to bring policy-makers, practitioners, and civil society together in the sense of transdisciplinary collaboration. Inter- and transdisciplinary work is essential to mobilize synergies, connect the different SDGs and enable science to be conducted with transformative action in mind. Those who have engaged in this type of venture know that disciplinary and institutional jargons are prime obstacles to well-functioning collaborations. In this regard the lessons learned from the PESTROP project (see **Table 1**), a research initiative bringing together five university institutes and non-governmental organizations (NGOs) were revealing. The study looking into the pesticide use in Costa Rica and Uganda combined insights from various disciplines and provided valuable insights into effective collaboration practices. The project showed that methodologically ‘modern’ approaches like stakeholder engagement processes, and design-thinking workshops were essential building blocks to develop scientifically solid and at the same time broadly anchored recommendations [16]. Overall, while such wider collaborations have transaction costs, they usually pay off in the longer term and the experiences from the PESTROP project confirm the 2030 Agenda for Sustainable Development’s insistence on inter- and transdisciplinary collaboration as a basis for consensual policy action.

## CHALLENGE #3: TIME LAG BETWEEN PROJECT IMPLEMENTATION AND MEASURABLE IMPACT

Potential solution: The time lag between the measurement and processing of indicators is an issue of concern. Two projects were presented at the symposium, showcasing advances in data management, processing, and analysis enabling the measurement of changes in health-related indicators in a timely manner (see **Table 1**). First, a precision global health approach, delivering near-real time disease surveillance and precision epidemic forecasting using digital technologies and artificial intelligence [17]. A second example pertained to risk profiling of malaria and other poverty-related diseases and spatially explicit impact measure of public health interventions [18].

## CHALLENGE #4: SUSTAINING IMPACT

Potential solution: To sustain impact on the ground and improve the health of the affected populations, researchers need to branch out from their usual publication focus and also aim for behavioural and policy change. Here, transdisciplinary approaches are highly effective, since they involve policy-makers from the onset thus facilitating research uptake once results become available. Even if a project is not transdisciplinary by design, researchers can still define a clear communication strategy and inform policy-makers about the evidence generated and the transformative pathways identified. Projects that approach a research topic from different angles, including natural sciences and engineering, life sciences and medicine, social sciences, gender studies and humanities, and law may be particularly suited to encourage behavioural and policy changes. For example (see **Table 1**), a promising approach is the inclusion of a governance stream, studying the policy context, as proposed in the PESTROP project [13]. Another example is the Dynamic Drivers of Disease in Africa Consortium (DDDAC), where health, environmental, social, and political science approaches were integrated, enabling the design of interventions that fitted the local social and political context and therefore promised more sustainable impact [19].

## CONCLUSION AND A WAY FORWARD

During this one-day symposium with a particular focus on health, several challenges in the implementation of the 2030 Agenda for Sustainable Development were identified and discussed. Most importantly, the SDGs provide a normative framework and offer potential solutions for sustainable development, including inter- and transdisciplinarity, open access and circulation of knowledge, and equitable partnerships. There is a pressing need to strengthen collaborations across disciplines, line ministries, and sectors, and between academics, practitioners, policy-makers, NGOs, and civil society, to tackle the SDGs in a more holistic manner. If we want to achieve the SDGs, we must transform the ways in which we work and we must do so quickly. Continuing with “business as usual” will fail to produce the relevant, timely, and impactful knowledge needed to achieve sustainable development.
